# Photosensitive Supramolecular Micelle-Mediated Cellular Uptake of Anticancer Drugs Enhances the Efficiency of Chemotherapy

**DOI:** 10.3390/ijms21134677

**Published:** 2020-06-30

**Authors:** Yihalem Abebe Alemayehu, Wen-Lu Fan, Fasih Bintang Ilhami, Chih-Wei Chiu, Duu-Jong Lee, Chih-Chia Cheng

**Affiliations:** 1 Graduate Institute of Applied Science and Technology, National Taiwan University of Science and Technology, Taipei 10607, Taiwan; yihalem2000@gmail.com (Y.A.A.); poi4515@yahoo.com.tw (W.-L.F.); fasihilhami17@gmail.com (F.B.I.); 2 Department of Materials Science and Engineering, National Taiwan University of Science and Technology, Taipei 10607, Taiwan; cwchiu@mail.ntust.edu.tw; 3 Department of Chemical Engineering, National Taiwan University of Science and Technology, Taipei 10607, Taiwan; djlee@ntu.edu.tw; 4 Department of Chemical Engineering, National Taiwan University, Taipei 10617, Taiwan; 5 Advanced Membrane Materials Research Center, National Taiwan University of Science and Technology, Taipei 10607, Taiwan

**Keywords:** chemotherapy, drug delivery, supramolecular micelle, photodimerization, uracil

## Abstract

The development of stimuli-responsive supramolecular micelles with high drug-loading contents that specifically induce significant levels of apoptosis in cancer cells remains challenging. Herein, we report photosensitive uracil-functionalized supramolecular micelles that spontaneously form via self-assembly in aqueous solution, exhibit sensitive photo-responsive behavior, and effectively encapsulate anticancer drugs at high drug-loading contents. Cellular uptake analysis and double-staining flow cytometric assays confirmed the presence of photo-dimerized uracil groups within the irradiated micelles remarkably enhanced endocytic uptake of the micelles by cancer cells and subsequently led to higher levels of apoptotic cell death, and thus improved the therapeutic effect in vitro. Thus, photo-dimerized uracil-functionalized supramolecular micelles may potentially represent an intelligent nanovehicle to improve the safety, efficacy, and applicability of cancer chemotherapy, and could also enable the development of nucleobase-based supramolecular micelles for multifunctional biomaterials and novel biomedical applications.

## 1. Introduction

Nano-sized drug delivery systems are widely used in pharmaceutical research and the clinic to enhance the therapeutic effects of anticancer drugs [1,2,3,4]. The use of nanocarriers can overcome several problems associated with traditional drugs, such as poor aqueous solubility, low bioavailability, and nonspecific distribution in the body [5,6]. Developments in materials science and pharmaceutics have enabled the design of various nanocarriers with diverse sizes, architectures, and surface properties that have potential as drug delivery systems, including polymers (polymeric micelles, nanoparticles, and dendrimers) [7,8], liposomes [9], inorganic materials [10,11], and metallic colloids [12]. Polymeric micelles have attracted attention as drug delivery systems because of their good biocompatibility, controlled self-assembly behavior, and high structural stability under physiological conditions [13,14,15,16]. In spite of these advantages, existing polymeric micelles possess a number of limitations, including inefficient drug-entrapment stability, low intracellular drug release rates, and modest accumulation in the target tissues [17]. Numerous publications have demonstrated that these challenges could potentially be overcome by modifying supramolecular polymeric micelles with well-defined hydrogen-bonding motifs [18,19,20]. Hydrogen bonding allows supramolecular polymer chains to rapidly self-assemble into materials with unique physical properties, such as controlled affinity, high specificity, and reversibility [21,22,23]. Most reported hydrogen-bonding mediated polymer assemblies have been designed to mimic the nucleobase paired nanostructures formed within RNA and DNA [24,25]. A variety of new biomaterials with specific properties have been synthesized from nucleobase-functionalized supramolecular polymers and other building blocks for specific biomedical applications such as bio-imaging, tissue engineering, gene therapy, and drug delivery [26,27,28]. The development of drug delivery systems that respond to specific stimuli is now a major research focus [26,28]. 

The creation of stimuli-responsive nanocarriers has opened the door to a new generation of anti-cancer drug delivery systems that are more intelligent and effective than conventional delivery systems [29,30]. Appropriately designed nanocarriers that respond to a particular stimulus, such as temperature, redox, light, magnetic field, or pH—or even a combination of two or more stimuli—may be able to intelligently target tumor tissues and thus improve treatment efficacy and reduce unwanted side effects in normal tissues [30,31,32,33,34]. Stimuli-responsive polymeric micelles exhibit enhanced accumulation in tumor tissues due to the enhanced permeability and retention effect. However, the instability and insufficient cellular internalization of drug-loaded micelles result in tumor cell drug dosages below the therapeutic window, which limits the induction of apoptosis in cancer cells [35,36]. These issues could potentially be overcome by functionalizing supramolecular polymers with physically and chemically cross-linkable moieties [37]. According to Adams [38] and Aliabadi [39], controlling hydrophobic effects within the inner micellar structure using chemical cross-links can enhance the stability of micelles. Enhancing micellar stability and increasing the hydrophobicity of supramolecular polymeric micelles could increase their cellular uptake and ability to induce apoptosis in cancer cells. The present work reports an in vitro evaluation of a novel polymeric micelle prepared from a physically and chemically cross-linked supramolecular polymer that functions as a carrier for anticancer drugs and improves the intracellular drug dosages and induction of apoptosis in cancer cells.

**Scheme 1:**
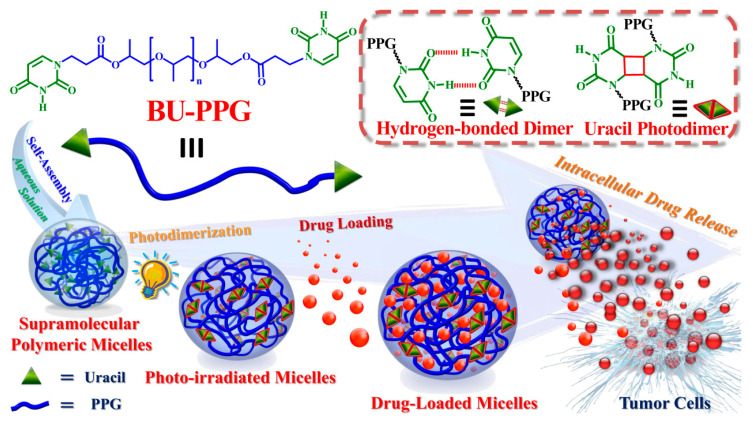
Schematic illustration of the formation of photosensitive uracil-functionalized BU-PPG micelles and drug release triggered in response to stimuli in the intracellular microenvironment of tumor cells.

In our previous studies, we synthesized an intelligent supramolecular polypropylene glycol (PPG) that possesses difunctional uracil-containing end groups (BU-PPG) by a simple one-step Michael addition reaction [40,41]. BU-PPG spontaneously assembles into thermo-sensitive nano-sized supramolecular micelles—both before and after photoirradiation—in aqueous solution. The micelles exhibit high structural stability, due to the presence of self-complementary hydrogen-bonding uracil moieties installed as a crosslinking element pendant to the polymeric backbone, and associate and dissociate in response to specific stimuli. Importantly, the photosensitive uracil moieties within the micelles undergo a [2 + 2] cycloaddition photoreaction, which facilitates supramolecular polymerization through chain extension of the end-functionalized PPG oligomers. Photodimerization enhances the stability, drug-loading capacity, and controlled drug release ability of the micelles. The tunable drug-loading capacity and controlled drug-release performance of these photosensitive BU-PPG micelles inspired us to evaluate their cytotoxic and therapeutic effects in cancer cells in vitro. The goal of this study is to achieve enhanced cellular internalization of photosensitive BU-PPG micelles to improve the safety of chemotherapy and therapeutic efficacy in cancer cells. Due to the presence of self-complementary uracil hydrogen bonding within the micelles, the drug-loaded BU-PPG micelles exhibit highly stable structures and excellent photo-responsive behavior in biological media. Importantly, compared to non-irradiated micelles, fluorescence imaging and flow cytometric analyses confirm the photo-dimerized uracil moieties substantially improved the uptake/intracellular accumulation of irradiated BU-PPG micelles and consequently enhanced the chemotherapeutic efficacy in tumor cells in vitro (Scheme 1). To the best of our knowledge, this is the first report of a supramolecular micelle based on hydrogen-bonded photo-dimerized uracil groups that is rapidly endocytosed by cancer cells and triggers high levels of apoptotic cell death. Thus, this self-complementary hydrogen-bonded and photo-crosslinked supramolecular micelle could potentially serve as a safe, effective nanocarrier to improve the chemotherapeutic effects of anticancer drugs.

## 2. Materials and Methods

The general materials, material preparations, and instrumentation used in this work are described in more detail in the Supplementary Material. 

## 3. Results and Discussion

The chemical structures of non-irradiated and irradiated BU-PPG polymers and their spontaneous self-organization into spherical nanosized micelles in water via formation of photo-dimerized uracil and hydrogen-bonded uracil dimers are presented in Scheme 1 [40,41]. Uracil end-capped difunctional oligomeric BU-PPG was successfully prepared by a simple one-step Michael addition reaction of PPG diacrylate (weight-average molecular weight of 800 g/mol, approximately 12 repeating units) to uracil, as described in our earlier report [40]. Doxorubicin (DOX) was selected as a chemotherapeutic drug and loaded into non-irradiated and irradiated BU-PPG micelles. Hydrogen bonding and π–π stacking interactions between the polar groups of the micelles and DOX molecules enhanced the retention and stability of DOX within the micelles and thereby conferred stable drug encapsulation ability and a wide-range tunable drug-loading content (DLC) [41]. The DLC, particle size distribution, and drug release behavior of the DOX-loaded non-irradiated and irradiated BU-PPG micelles in phosphate-buffered saline (PBS) were discussed in detail in our previous report [41]. Here, we briefly describe photodimerization and drug encapsulation within the polymeric micelles. During ultraviolet (UV) irradiation (254 nm, 50−70 mW/cm^2^), the intensity of the C=C double bond absorbance peak at 263 nm in the UV-Vis spectra of BU-PPG solution gradually decreased due to the [2π + 2π] photocycloaddition reaction between uracil groups. The peak eventually leveled off after 60 min irradiation (Figure S1) [42], indicating BU-PPG was almost completely photo-dimerized (calculated conversion efficiency of 95%), in good agreement with previous experimental results (96%) [40]. After encapsulating DOX at a 1:1 polymer/drug weight ratio by a dialysis method, the average particle diameters of irradiated and non-irradiated BU-PPG micelles increased from 141 nm and 148 nm to 180 nm and 189 nm, with DLC values of 24.6% and 18.4%, respectively (Figure S2 and Table S1). Moreover, the zeta potential values of DOX-loaded non-irradiated and irradiated BU-PPG micelles in PBS increased to 30.1 and 34.2 mV, respectively, compared to 1.4 mV for non-irradiated micelles and 14.9 mV for irradiated micelles [41]. Thus, irradiation increases the solubility and enhances the overall structural stability of the DOX-loaded micelles (Table S1). These results prompted us to assess the cytotoxicity of non-irradiated and irradiated DOX-loaded BU-PPG micelles in vitro.

We hypothesized that DOX-loaded BU-PPG micelles would enable selective drug release in the acidic intracellular tumor microenvironment and improve the overall therapeutic efficacy of cancer chemotherapy. The cytotoxicity of non-irradiated and irradiated BU-PPG micelles and DOX-loaded micelles against non-cancerous NIH/3T3 fibroblast cells (a mouse embryonic fibroblast cell line) and HeLa cells (a human cervical cancer cell line) was evaluated using the methyl blue thiazol tetrazolium (MTT) assay. No significant changes in cell viability were observed after 24 h incubation with a wide range of concentrations of non-irradiated or irradiated BU-PPG micelles at 37 °C and pH 7.4, even at high micelle concentrations of up to 100 µg/mL (Figures S3 and S4). These results indicate that the non-irradiated and irradiated BU-PPG micelles are highly biocompatible and have potential as an anticancer drug nanocarrier. However, the viability of HeLa cells gradually decreased as the concentration of DOX encapsulated within the non-irradiated and irradiated BU-PPG micelles was increased (Figure S5), suggesting that the encapsulated DOX reduced the cell viability of HeLa cells in a concentration-dependent manner. Moreover, these results indicate DOX was released from the micelles due to rapid dissociation of the hydrogen-bonded uracil dimers in the acidic intracellular tumor microenvironment [43]. Surprisingly, DOX-loaded irradiated BU-PPG micelles exhibited a lower half maximal inhibitory concentration (IC_50_) value (4.12 ± 0.22 µg/mL) than DOX-loaded non-irradiated micelles (4.42 ± 0.30 µg/mL), suggesting the irradiated BU-PPG micelles can more rapidly cross the tumor cell membranes, where the acidic intracellular tumor environment subsequently triggers DOX release from the micelles, which induces programmed cell death. Another possible factor could be the higher thermoresponsive behavior of irradiated BU-PPG micelles, which confers a more rapid DOX release rate, compared to DOX-loaded non-irradiated micelles [41]. However, both irradiated and non-irradiated DOX-loaded micelles had slightly higher IC_50_ values than free DOX (3.71 ± 0.14 µg/mL); in the absence of the micelles as a drug delivery carrier, DOX can exert significant cytotoxic effects in normal surrounding cells [44]. Overall, these observations demonstrate that DOX-loaded irradiated BU-PPG micelles more rapidly enter and release DOX in the acidic intracellular microenvironment of tumor cells, which results in higher toxicity, compared to DOX-loaded non-irradiated micelles. 

**Figure 1:**
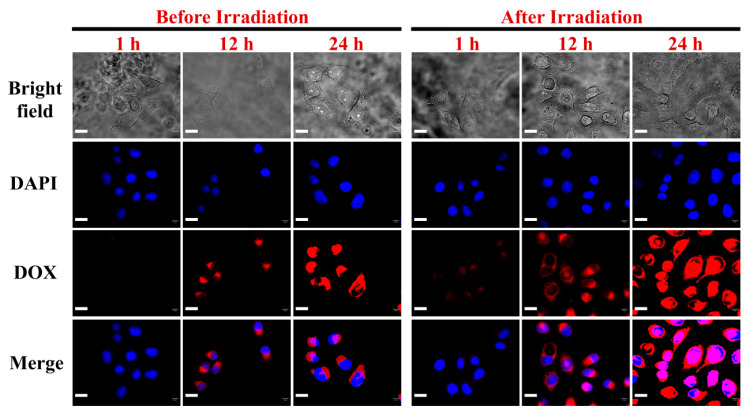
Confocal laser scanning microscopy (CLSM) images of HeLa cells incubated with non-irradiated and irradiated DOX-loaded BU-PPG micelles. Cell nuclei were stained with 4′,6-diamidino-2-phenylindole (DAPI, blue fluorescence); red signals indicate the characteristic fluorescence of DOX. Top and middle panels display the bright-field, blue-, and red-colored fluorescent images, respectively; the lower panel is the merged image of blue DAPI fluorescence and red DOX fluorescence. Scale bar for all images is 20 μm.

To obtain further insight into the cytotoxicity of DOX-loaded non-irradiated and irradiated BU-PPG micelles, we assessed cellular uptake using confocal laser scanning microscopy (CLSM) and flow cytometry. The distributions of DOX-loaded non-irradiated and irradiated micelles (4 μg/mL) were examined in HeLa cells after 1, 12, and 24 h incubation using CLSM. As demonstrated in Figure 1, DOX-loaded non-irradiated and irradiated BU-PPG micelles were only localized in the cytoplasm (not the nuclei) after 12 h. These results indicate both the non-irradiated and irradiated DOX-loaded micelles were rapidly internalized into the cancer cells, and delayed the entry of DOX into the nucleus. However, after 24 h, DOX fluorescence was clearly distributed inside both the cytoplasm and nuclei of the tumor cells incubated with DOX-loaded non-irradiated and irradiated micelles, indicating the DOX-loaded micelles were rapidly taken up and gradually accumulated in the nuclei as the incubation time increased. Regardless of the length of the incubation period, higher levels of DOX fluorescence intensity were observed in HeLa cells treated with irradiated micelles than non-irradiated micelles, suggesting the irradiated micelles were more sensitive to the acidic intracellular tumor environment and thus released DOX more rapidly. This also indicates the DOX-loaded irradiated micelles entered the tumor cells more rapidly than non-irradiated micelles, possibly due to a high-affinity interaction between the irradiated micelles and HeLa cells [43], thus DOX-loaded irradiated micelles could effectively induce massive cell death in tumor cells. These results also imply that the photo-dimerized uracil moieties of the irradiated micelles enhance cellular uptake of irradiated supramolecular BU-PPG micelles by HeLa cells. 

**Figure 2:**
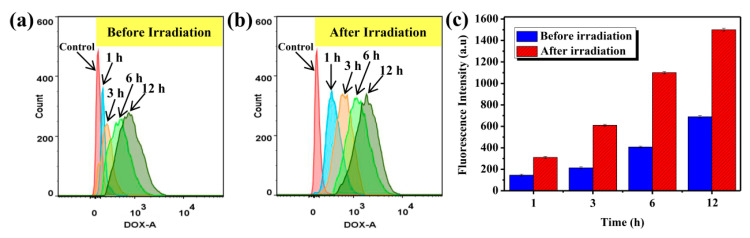
Flow cytometric profiles of HeLa cells treated with non-irradiated (**a**) and irradiated (**b**) DOX-BU-PPG micelles. (**c**) Fluorescence intensity of HeLa cells after incubation with DOX-loaded non-irradiated and irradiated micelles for various periods of time.

Next, we confirmed these observations using quantitative flow cytometry analysis. As shown in Figure 2a,b, significant increases in DOX fluorescence intensity were observed in HeLa cells treated with irradiated and non-irradiated DOX-loaded BU-PPG micelles as the incubation time increased, suggesting uptake of the DOX-loaded micelles by HeLa cells increased with the incubation period. Much stronger DOX fluorescence was clearly observed in cells incubated with the irradiated BU-PPG micelles than non-irradiated micelles; this effect could possibly be attributed to specific interactions between HeLa cells and the photo-dimerized uracil moieties of the irradiated micelles, which may enhance internalization of the irradiated micelles in HeLa cells. These flow cytometry results were consistent with the CLSM data, and further confirm that supramolecular polymerization of BU-PPG through photoirradiation improves the cellular uptake of the micelles by cancer cells. This rare, highly desirable feature is required to improve the chemotherapeutic efficacy of functional drug nanocarrier systems. Next, we assessed how photoirradiation affects the cellular uptake of DOX-loaded micelles by plotting the average fluorescence intensity of DOX versus incubation time. As shown in Figure 2c, the rate of cellular uptake of DOX-loaded irradiated micelles was approximately 2.2 times higher than that of DOX-loaded non-irradiated micelles in HeLa cells treated for up to 12 h. However, the exact mechanism that enhances the cellular uptake of photo-irradiated micelles is still unknown. Thus, we conducted a quantitative study of cell death in cells treated with irradiated and non-irradiated DOX-loaded micelles to further investigate the significantly higher cellular uptake of photo-dimerized DOX-loaded micelles by HeLa cells.

**Figure 3:**
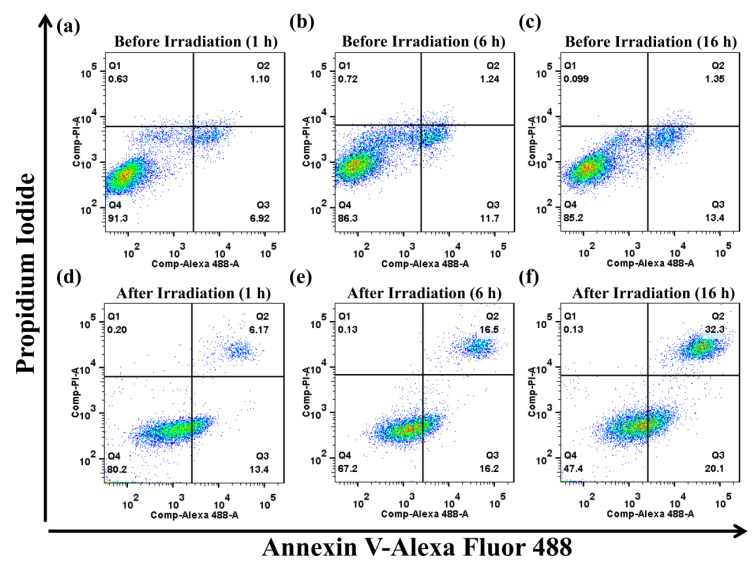
Flow cytometric analysis of apoptosis in HeLa cells treated with DOX loaded (**a**–**c**) non-irradiated and (**d**–**f**) irradiated BU-PPG micelles for 1, 6, and 16 h. Region Q1 represents necrotic cells (Annexin−/PI+); Q2, late apoptotic cells (Annexin+/PI+); Q3, early apoptotic cells (Annexin+/ PI−); Q4, living cells (Annexin−/PI−). The numbers in the profiles indicate the percentages of cells in each quadrant.

In order to clarify the apoptotic pathway and endocytic mechanisms underlying the chemotherapeutic effects of photosensitive supramolecular micelles, HeLa cells were treated with DOX-loaded non-irradiated or irradiated BU-PPG micelles for 1, 6, or 16 h. Annexin V-Alexa Fluor488 (Annexin V)/propidium iodide (PI) double-staining was used to identify and quantify apoptosis/necrosis by flow cytometry. As shown in Figure 3, surprisingly, the percentage of HeLa cells undergoing early/late apoptosis was significantly higher for cells incubated with DOX-loaded irradiated BU-PPG micelles than DOX-loaded non-irradiated BU-PPG micelles for the same period of time under normal physiological conditions (37 °C and pH 7.4). These results were consistent with the CLSM images and flow cytometry. As shown in Figure 3c, the total percentage of early/late apoptotic cells was less than 15% (13.4% early apoptotic cells and 1.4% late apoptotic cells) for HeLa cells incubated with DOX-loaded non-irradiated BU-PPG micelles for 16 h, indicating that the DOX-loaded non-irradiated micelles were not rapidly transported into the HeLa cells and only induced low levels of early/late apoptosis. In contrast, the relative percentages of early/late apoptotic cells were substantially higher (20.1% and 32.3%) in HeLa cells incubated with DOX-loaded irradiated micelles for 16 h, while necrotic cells were almost undetectable (0.13%; Figure 3f). This further confirms the photo-dimerized uracil group’s promote rapid cellular uptake of irradiated DOX-loaded micelles by HeLa cells through endocytosis, and that the acidic intracellular tumor microenvironment subsequently promotes rapid release of the encapsulated DOX and induces high levels of apoptotic cell death. Moreover, these results also indicate DOX-loaded irradiated micelles induce apoptosis in a time-dependent manner: higher numbers of apoptotic cells were observed after a longer incubation period, which could be attributed to rapid destruction of the self-assembled micelles due to dissociation of the self-complementary hydrogen bonding interactions between the photo-dimerized uracil moieties. Overall, this study demonstrates that photo-dimerized uracil-functionalized BU-PPG micelles could potentially represent an intelligent nanocarrier with the potential to improve the efficacy, safety, and applicability of chemotherapy, and may also enable the development of nucleobase-based polymeric micelles as multifunctional biomaterials for various biomedical applications.

## 4. Conclusions

We reported the design of photosensitive BU-PPG polymers containing two uracil end-groups that overcome numerous issues related to existing nanocarriers. Owing to the presence of photo-dimerized uracil and hydrogen-bonded uracil dimers in the polymer structure, BU-PPG spontaneously self-organizes into spherical nanosized micelles in water and can encapsulate DOX at a high drug-loading content. *In vitro* cytotoxicity and fluorescence imaging studies indicated the DOX-loaded irradiated micelles were rapidly and efficiently taken up into cancer cells and led to a significantly greater reduction in cell viability and higher proportions of apoptotic cells than non-irradiated micelles. Annexin V/PI-staining and flow cytometric analysis further demonstrated that the presence of the photo-dimerized uracil moieties within the polymeric structures facilitated rapid cellular internalization of the irradiated micelles by HeLa cells. Moreover, the acidic intracellular environment significantly accelerated the release of encapsulated DOX and subsequently triggered high levels of cell death via apoptosis. Thus, the presence of the photo-dimerized uracil moieties within the irradiated micelles enhanced the cellular uptake of therapeutic drugs and led to high levels of apoptosis in tumor tissues under normal physiological conditions. Overall, these photosensitive uracil-based micelles represent a promising multifunctional drug delivery nanovector that may potentially improve the safety and efficacy of cancer chemotherapy.

## Supplementary Materials

The following are available online at https://www.mdpi.com/1422-0067/21/13/4677/s1, Figure S1: UV-Vis absorbance spectra of BU-PPG (0.04 mg/mL in aqueous solution irradiated for 60 min at 254 nm). The inset figure illustrates the photoirradiation reaction of the uracil moieties in BU-PPG, Figure S2: DLS of free BU-PPG micelles before and after irradiation and DOX-loaded BU-PPG micelles before and after irradiation, Figure S3: Cytotoxicity of non-irradiated BU-PPG micelles towards NIH/3T3 cells after 24 h incubation, Figure S4: Cytotoxicity of irradiated BU-PPG micelles towards HeLa cells after 24 h incubation, Figure S5: Cytotoxicity of free DOX, DOX-loaded non-irradiated, and DOX-loaded irradiated BU-PPG micelles towards HeLa cells after 24 h incubation, Table S1: Particle size, zeta potential, drug loading efficiency, and drug loading content of DOX-loaded BU-PPG micelles.
