# Supplementary material for: Photosensitive Supramolecular Micelle-Mediated Cellular Uptake of Anticancer Drugs Enhances the Efficiency of Chemotherapy
Source: Int J Mol Sci. 2020 Jun 30;21(13):4677. doi: 10.3390/ijms21134677 (PMC7370087; doi:10.3390/ijms21134677)
Supplement: Supplementary file 1 [file ijms-21-04677-s001.pdf]

# Supplementary Materials

## Photosensitive Supramolecular Micelle-Mediated Cellular Uptake of Anticancer Drugs Enhances the Efficiency of Chemotherapy

Yihalem Abebe Alemayehu,[a] Wen-Lu Fan,[a] Fasih Bintang Ilhami[a] Chih-Wei Chiu,[c] Duu-Jong Lee[d,e] and Chih-Chia Cheng[a,b]*

a. Graduate Institute of Applied Science and Technology, National Taiwan University of Science and Technology, Taipei 10607, Taiwan. E-mail: cccheng@mail.ntust.edu.tw

b. Advanced Membrane Materials Research Center, National Taiwan University of Science and Technology, Taipei 10607, Taiwan.

c. Department of Materials Science and Engineering, National Taiwan University of Science and Technology, Taipei 10607, Taiwan.

d. Department of Chemical Engineering, National Taiwan University of Science and Technology, Taipei 10607, Taiwan.

e. Department of Chemical Engineering, National Taiwan University, Taipei 10617, Taiwan.

* Corresponding Author

## Experimental

### *Materials*

Poly(propylene glycol) diacrylate (average molecular weight: ~800 g/mol), uracil, dimethylformamide (DMF), potassium tertbutoxide were purchased at the highest purity available from Sigma-Aldrich (St. Louis, MO, USA) and Acros Organics (Geel, Belgium). All other highly purified chemicals and reagents were purchased from Sigma-Aldrich. Material synthesis and characterization are described in detail in our previous studies [40,41].

### *Photo-dimerization of BU-PPG aqueous solution*

BU-PPG was irradiated using an ultraviolet curing instrument (Xlite 500, OPAS, Taiwan; light source, 254 nm, irradiation intensity, 50-70 mW/cm$^2$, at a sample distance of 8 cm from the UV lamp for 60 min). The degree of polymerization was monitored by measuring the absorbance of BU-PPG before and after irradiation using an ultraviolet-visible (UV-Vis) spectrophotometer (Jasco V-730, Hitachi, Tokyo, Japan).

### *Preparation of DOX-loaded micelles*

Doxorubicin hydrochloride (DOX·HCl) was mixed with a three molar equivalent of triethylamine in 2 mL of DMF and stirred for 30 min. DOX-loaded irradiated and non-irradiated BU-PPG polymeric micelles were prepared by a dialysis method. In brief, the polymer and doxorubicin were dissolved in 2 mL DMF at a 1:1 weight ratio, 10 mL ultra-purified water (Millipore, 18.2 MΩ·cm) was added gradually while stirring the solution moderately, the mixture was stirred for 24 h, and DMF was removed by dialysis against water to obtain DOX-loaded micelles.

The drug loading capacity of irradiated and non-irradiated BU-PPG micelles was determined by UV-Vis spectrophotometry. Briefly, 2 mg of lyophilized nanoparticles were

dissolved in water, and drug loading content (DLC) and loading efficiency (DLE) were calculated using the following equations.

$$\text{DLC}\ (\%) = \frac{\text{Weight of drug in micelles}}{\text{Weight of drug loaded micelles}}\ \text{x}\ 100$$

$$\text{DLE}\ (\%) = \frac{\text{Weight drug in micelles}}{\text{Weight of the drug imput}}\ \text{x}\ 100$$

***Cell culture***

HeLa and NIH/3T3 cells were cultured in Dulbecco's modified eagle medium (DMEM) containing 10% fetal bovine serum (FBS) and 1% antibiotics (penicillin-streptomycin, 10,000 U/mL) in a humidified chamber at 37 °C in a 5% $CO_2$ atmosphere.

***In vitro cytotoxicity assays***

To evaluate the effect of the DOX concentration on cell viability, the cytotoxicity of DOX–loaded BU-PPG micelles were assessed using the MTT assay. Briefly, 1× $10^6$ cells per well were seeded into 96-well plates in 100 μL DMEM. After 24 h, the media was removed, and 100 μL DMEM containing different concentrations of DOX (ranging from 0.01 to 100 μg/mL) was added, incubated at 37 °C for 24 h, then 20 μL of MTT solution in PBS (5 mg/mL) was added to each well, incubated for 4 h, the MTT-generated formazan was dissolved in 150 μL DMSO per well, and absorbance was determined using an ELISA microplate reader (Thermo Fisher Scientific, Waltham, MA, USA) at a wavelength of 570 nm. All results are reported as the mean and standard deviation (± SD) of triplicate measurements. $IC_{50}$ values were determined by plotting cell viability (%) versus DOX concentration (μg/mL). The same technique was used to determine the toxicities of blank BU-PPG micelles (before and after irradiation) towards HeLa and NIH/3T3 cells.

***Cellular uptake***

HeLa cells were seeded into 6-well plates ($5 \times 10^5$ cells/well) in 2 mL complete DMEM media, incubated for 24 h, and the culture media was replaced with 2 mL fresh media containing DOX-loaded BU-PPG micelles (irradiated or non-irradiated) at 4 μg/mL DOX. The cells were cultivated for 1, 12, or 24 h at 37 °C, fixed in paraformaldehyde (4%) for 30 min and stained with 4′,6-diamidino-2-phenylindole (DAPI) for 5 min to visualize the nuclei. Confocal laser scanning microscopy (CLSM) images were obtained using an Olympus FluoView 1000 (Tokyo, Japan).

Cellular uptake of various formulations was confirmed by flow cytometry. Briefly, HeLa cells were seeded into six-well plates ($5 \times 10^5$ cells per well) in 2 mL complete DMEM media, incubated for 24 h, and treated with DOX or irradiated and non-irradiated DOX-loaded BU-PPG micelles for 1, 3, 6 or 12 h at 37 °C. Cellular fluorescence was measured by flow cytometry (FACScan; Becton Dickinson, Heidelberg, Germany).

***Cell apoptosis***

HeLa cells were seeded in 6-well plates ($5.0 \times 10^6$ cells per well) in 2 mL complete DMEM, cultured for 24 h, treated with DOX-loaded BU-PPG micelles for 1, 6 or 16 h, harvested, and stained with Annexin V and PI for 15 min at 37 °C in the dark. Flow cytometry (FACScan; Becton Dickinson, Heidelberg, Germany) was used to quantify apoptosis; $1 \times 10^4$ events were counted per sample.

***Statistical analysis***

All data are the average values ± SD of at least three independent experiments.

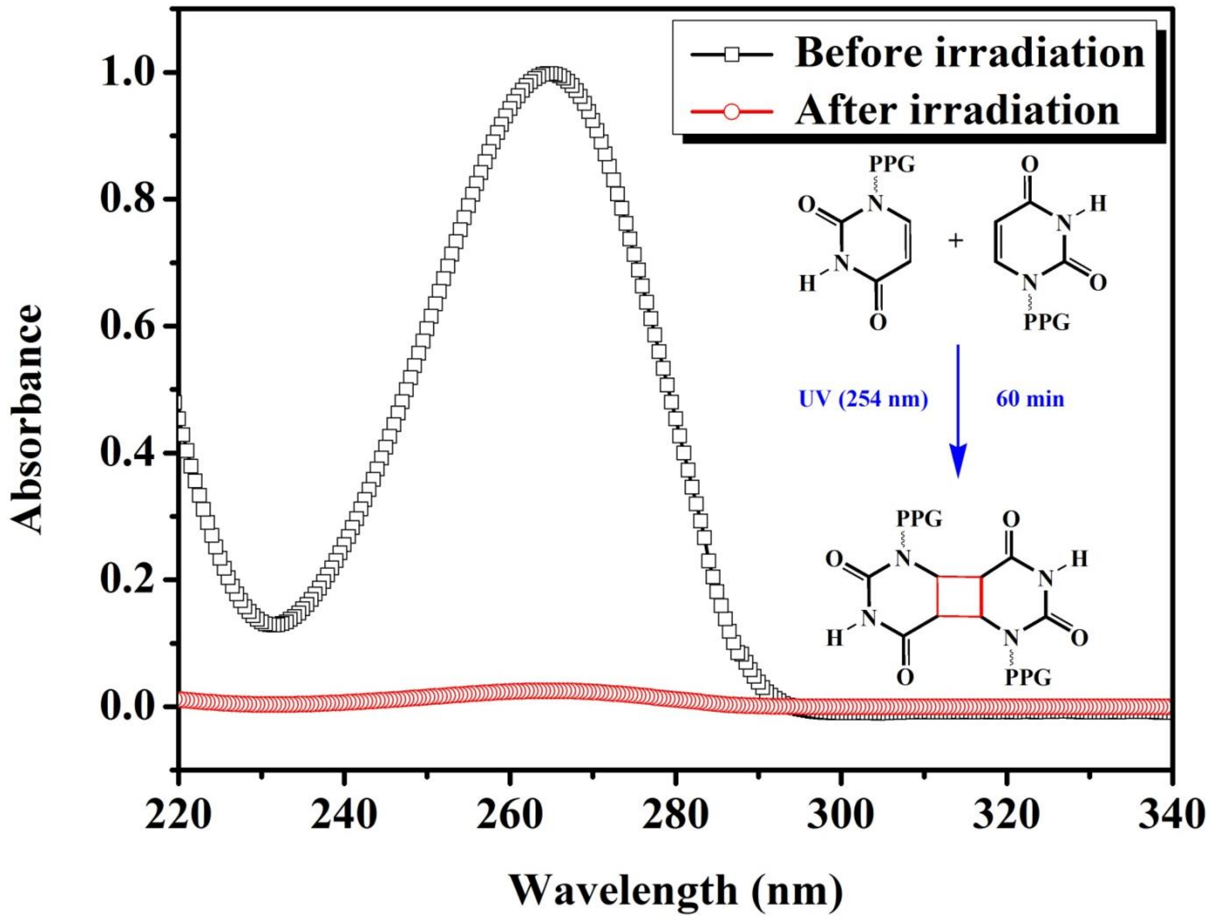


**Figure S1:** UV-Vis absorbance spectra of BU-PPG (0.04 mg/mL in aqueous solution irradiated for 60 min at 254 nm). The inset figure illustrates the photoirradiation reaction of the uracil moieties in BU-PPG.

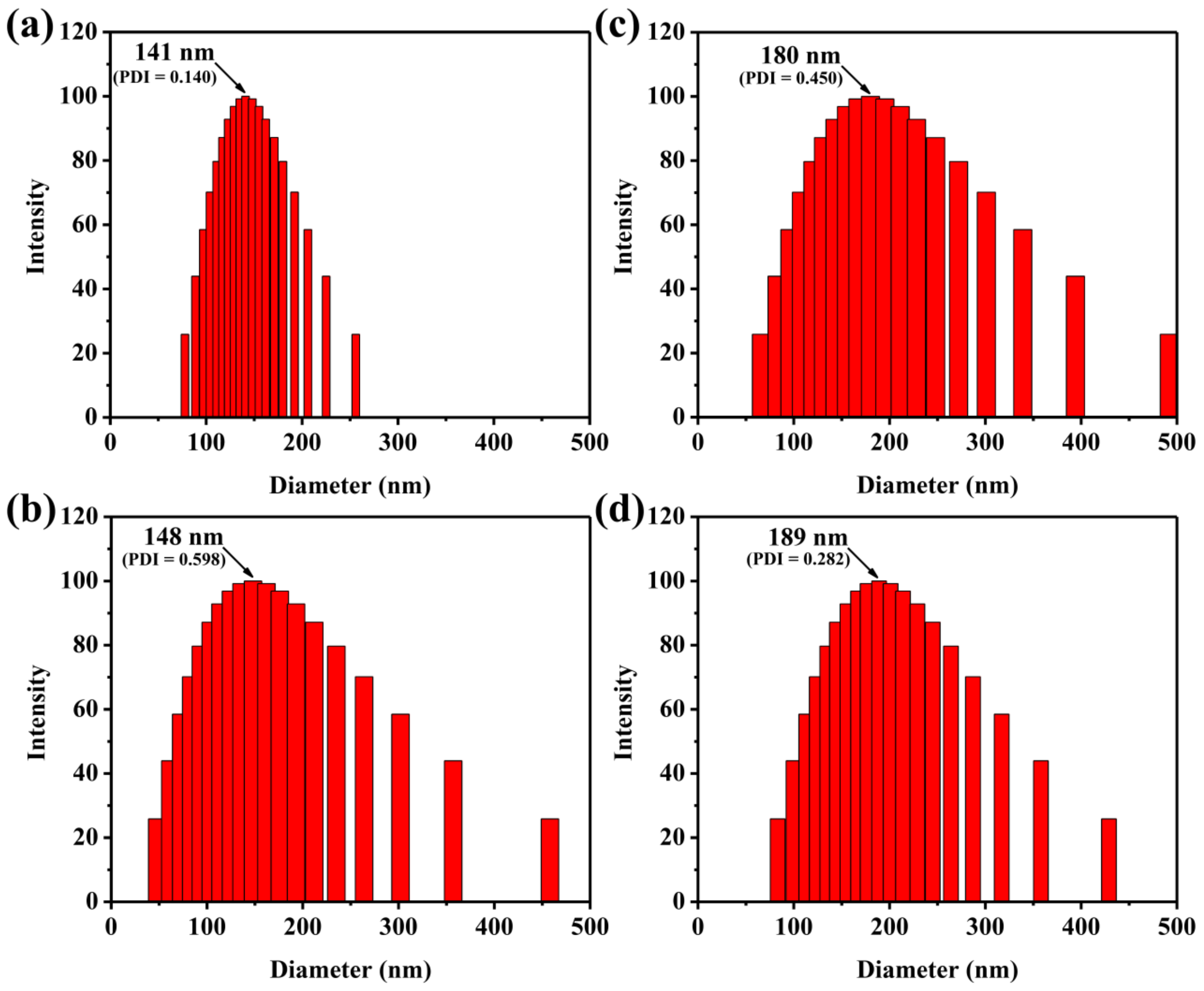


**Figure S2:** DLS of free BU-PPG micelles **(a)** before and **(b)** after irradiation and DOX-loaded BU-PPG micelles before **(c)** and after **(d)** irradiation.

**Table S1:** Particle size, zeta potential, drug loading efficiency (DLE) and drug loading content (DLC) of DOX-loaded BU-PPG micelles.

| Drug-loaded material | DOX:BU-PPG weight ratio | Diameter ± SD (nm) | PDI ± SD[2] | Zeta potential ± SD (mV) | DLC ± SD[3] (%) | DLE ± SD[4] (%) |
|---|---|---|---|---|---|---|
| After irradiation | 1:1 | 189 ± 10.28 | 0.598 ± 0.023 | 34.24 ± 1.64 | 24.61 ± 0.55 | 65.30 ± 2.11 |
| Before irradiation | 1:1 | 180 ± 8.45 | 0.140 ± 0.011 | 30.13 ± 1.52 | 18.37 ± 0.86 | 45.02 ± 1.53 |

[1] The micelle concentration in the initial PBS solution was 3.0 mg/mL. Measurements were performed in phosphate buffered saline (PBS; pH 7.4, 10 mM); particle size was measured by DLS.

[2] PDI = polydispersity index, which represents the width of the particle size distribution. Values are mean ± standard deviation, $n = 3$ or 10.

[3] DLC = drug loading content.

[4] DLE = drug loading efficiency.

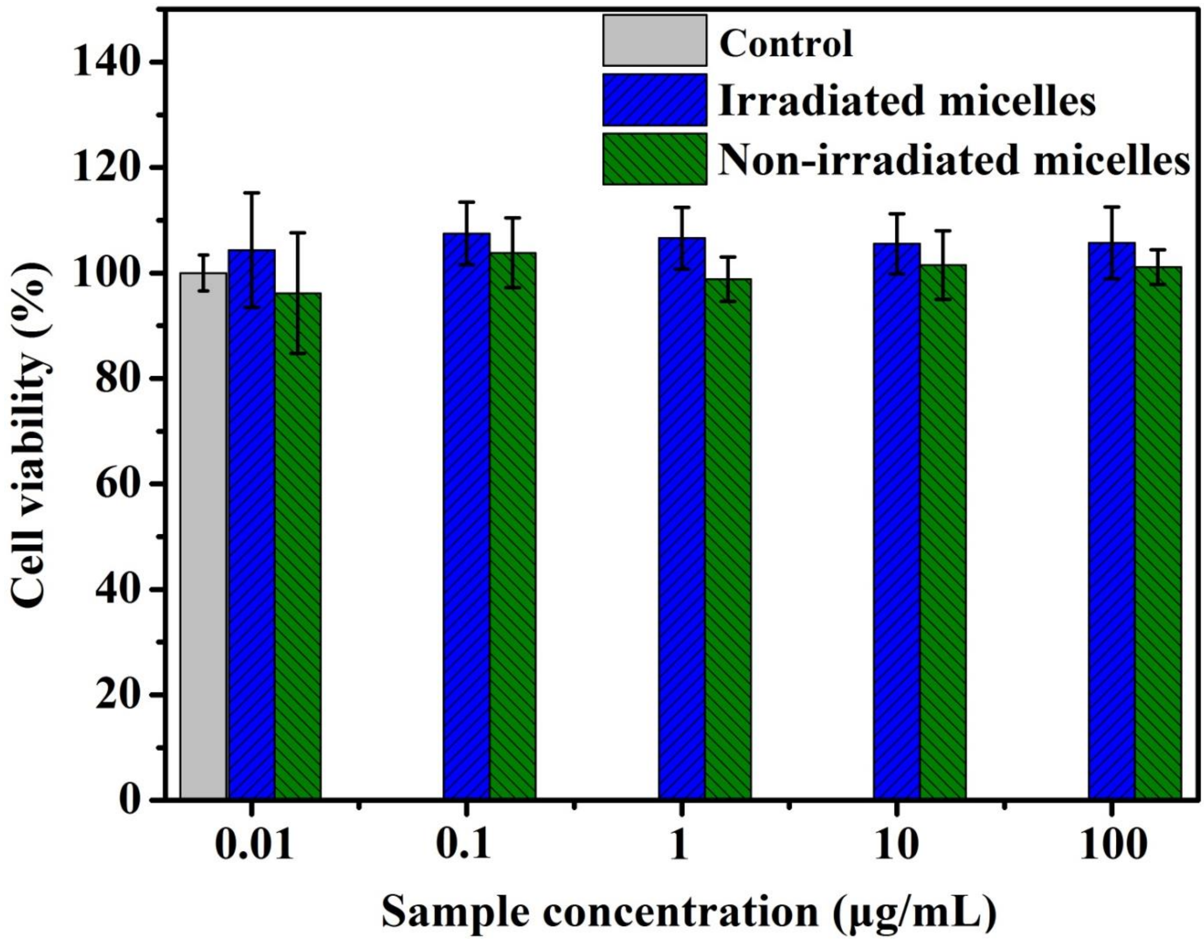


**Figure S3:** Cytotoxicity of non-irradiated and irradiated BU-PPG micelles towards NIH/3T3 cells after 24 h incubation.

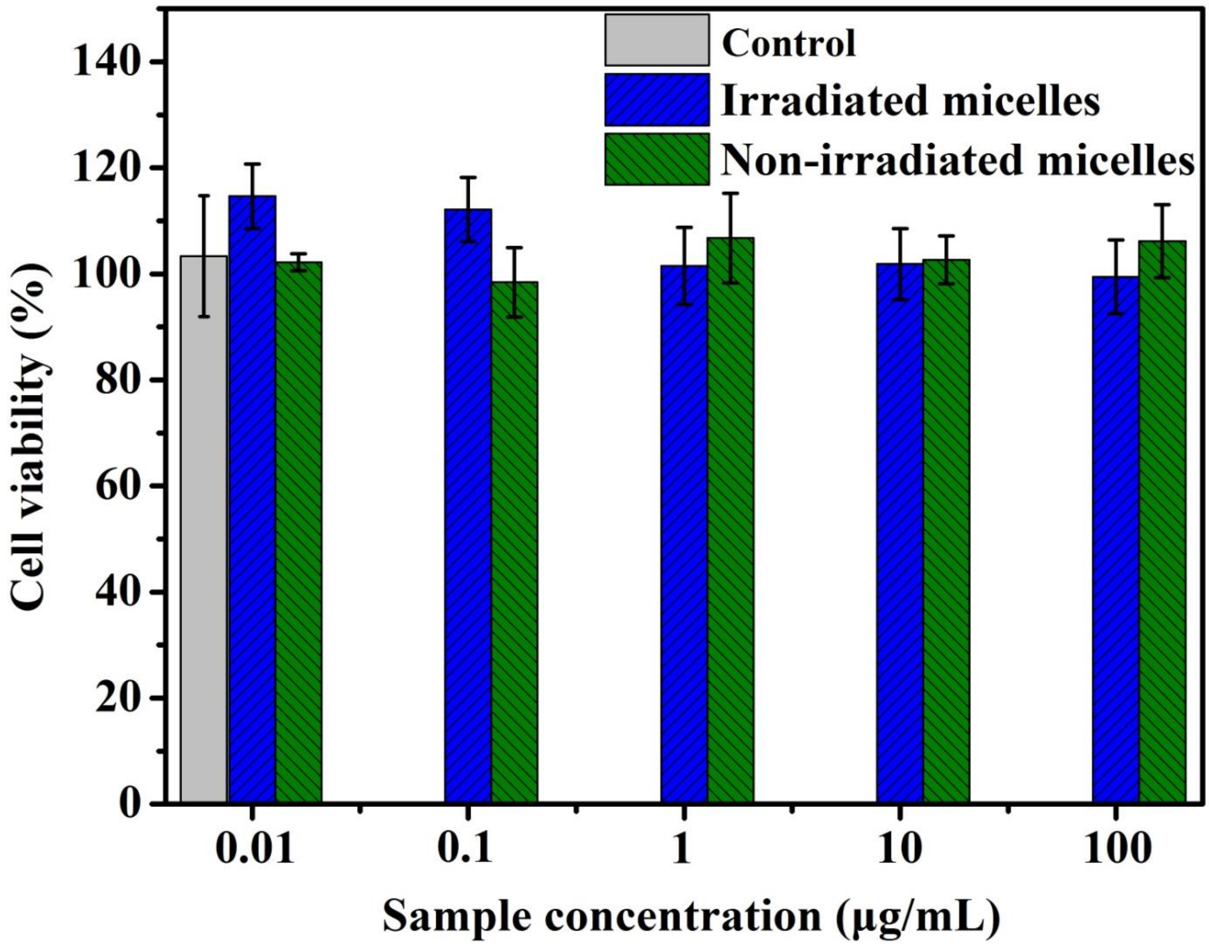


**Figure S4:** Cytotoxicity of non-irradiated and irradiated BU-PPG micelles towards HeLa cells after 24 h incubation.

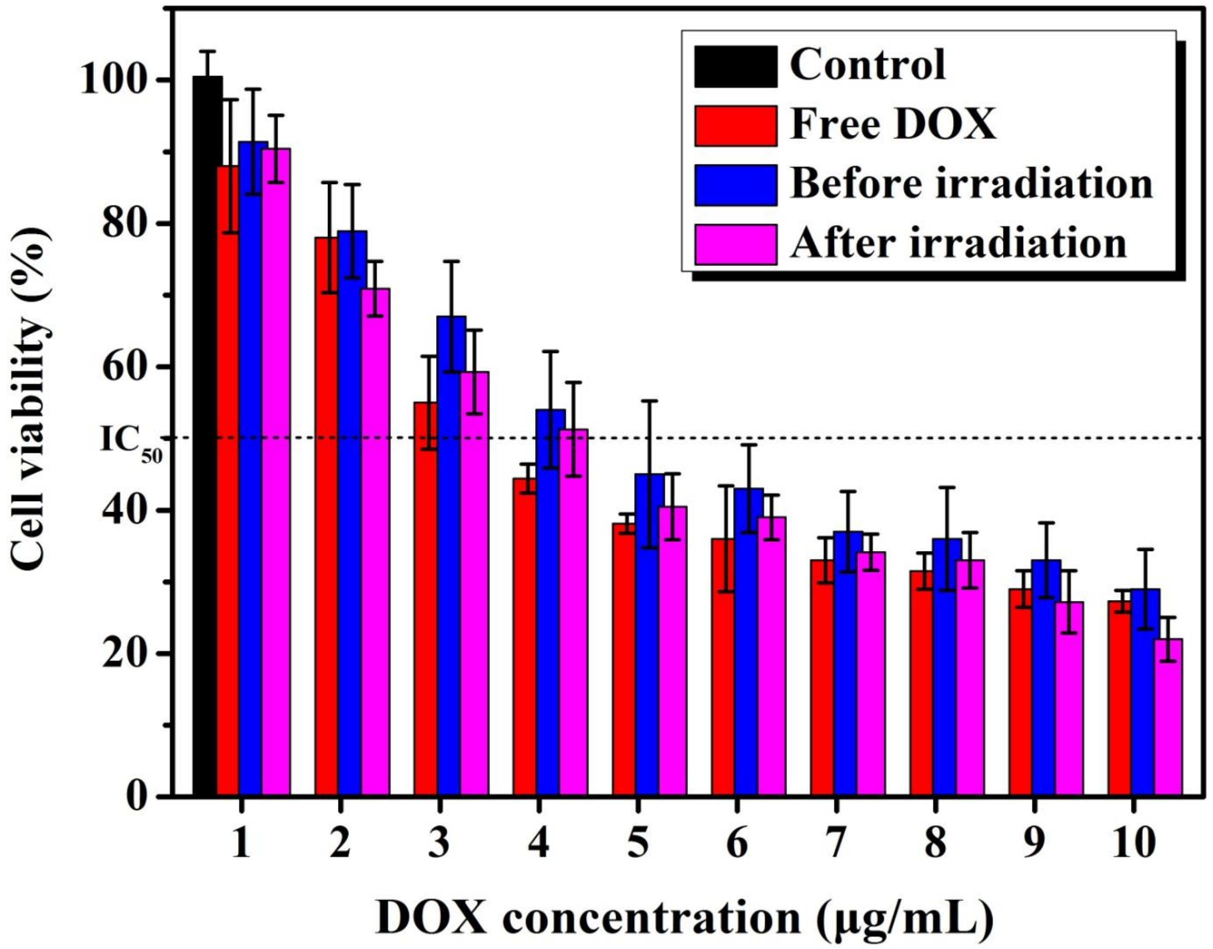


**Figure S5:** Cytotoxicity of free DOX, DOX-loaded non-irradiated and DOX-loaded irradiated BU-PPG micelles towards HeLa cells after 24 h incubation.
